# Rule discovery and distance separation to detect reliable miRNA biomarkers for the diagnosis of lung squamous cell carcinoma

**DOI:** 10.1186/1471-2164-15-S9-S16

**Published:** 2014-12-08

**Authors:** Renhua Song, Qian Liu, Gyorgy Hutvagner, Hung Nguyen, Kotagiri Ramamohanarao, Limsoon Wong, Jinyan Li

**Affiliations:** 1 Advanced Analytics Institute, University of Technology, Sydney, Broadway New South Wales 2007, Australia; 2 Centre for Health Technologies, University of Technology, Sydney, Broadway New South Wales 2007, Australia; 3 Department of Computing and Information Systems, the University of Melbourne, Victoria 3010, Australia; 4 School of Computing, National University of Singapore, Singapore 117417,Singapore

## Abstract

**Background:**

Altered expression profiles of microRNAs (miRNAs) are linked to many diseases including lung cancer. miRNA expression profiling is reproducible and miRNAs are very stable. These characteristics of miRNAs make them ideal biomarker candidates.

**Method:**

This work is aimed to detect 2-and 3-miRNA groups, together with specific expression ranges of these miRNAs, to form simple linear discriminant rules for biomarker identification and biological interpretation. Our method is based on a novel committee of decision trees to derive 2-and 3-miRNA 100%-frequency rules. This method is applied to a data set of lung miRNA expression profiles of 61 squamous cell carcinoma (SCC) samples and 10 normal tissue samples. A distance separation technique is used to select the most reliable rules which are then evaluated on a large independent data set.

**Results:**

We obtained four 2-miRNA and three 3-miRNA top-ranked rules. One important rule is that: If the expression level of miR-98 is above 7.356 and the expression level of miR-205 is below 9.601 (log2 quantile normalized MirVan miRNA Bioarray signals), then the sample is normal rather than cancerous with specificity and sensitivity both 100%. The classification performance of our best miRNA rules remarkably outperformed that by randomly selected miRNA rules. Our data analysis also showed that miR-98 and miR-205 have two common predicted target genes FZD3 and RPS6KA3, which are actually genes associated with carcinoma according to the Online Mendelian Inheritance in Man (OMIM) database. We also found that most of the chromosomal loci of these miRNAs have a high frequency of genomic alteration in lung cancer. On the independent data set (with balanced controls), the three miRNAs miR-126, miR-205 and miR-182 from our best rule can separate the two classes of samples at the accuracy of 84.49%, sensitivity of 91.40% and specificity of 77.14%.

**Conclusion:**

Our results indicate that rule discovery followed by distance separation is a powerful computational method to identify reliable miRNA biomarkers. The visualization of the rules and the clear separation between the normal and cancer samples by our rules will help biology experts for their analysis and biological interpretation.

## Background

miRNAs are a class of small (19-25 nucleotides) and endogenous non-coding RNAs which play important roles in various biological processes [1-7]. For example, miRNAs can regulate gene expression at the post-transcriptional stage, and can control fundamental cellular processes such as differentiation, cell growth, proliferation and apoptosis [1,3,4,7,8]. In fact, miRNAs have the potential to regulate at least 20-30% of all human transcripts [6,9-11]. They have also been shown to control the expression of oncogenes and tumor-suppressor genes [4,12-14]. Aberrant miRNA expressions have been linked to many diseases, and have been intensively investigated recently to discover miRNA biomarkers for the diagnosis of diseases including lung cancer [10,15-17]. The inherent stability of miRNAs in serum and the reliability and reproducibility of expression analysis [5,9,18-21] make them ideal candidates for biomarkers [22].

However, recent studies have often focused on statistical and biological significance of single miRNAs by identifying differentially expressed individual miRNAs as biomarkers [23]. The problem is that single-miRNA rules are insufficient for accurate diagnosis [24]. For example, Raponi *et al*. [10] identified 15 miRNAs differentially expressed between normal and squamous cell carcinoma (SCC) samples. None of them has good sensitivity. This is probably because target mRNAs are actually affected simultaneously by multiple miRNAs [25,26] synergistically or possibly several miRNAs-regulated pathways are involved in the progression of the disease [27].

Lung cancer is often diagnosed at a late stage with poor prognosis [27,28]. It is also the leading cause of cancer-related deaths worldwide [27]. Non-small cell lung cancers (NSCLC) are the major types of lung cancer, comprised mainly of adenocarcinoma and SCC. Algorithms to diagnose early-stage SCC are vital for improving the survival rate of the patients [29]. Chest X-ray has been applied for its early detection, but it has low sensitivity [30-33]. Other studies have identified gene mutation spectra and gene expression profiles associated with biological processes that are altered in lung cancer [3,34], resulting in improved sensitivity. As miRNAs are promising biomarker candidates [35], we specially use miRNA groups to form simple and strong rules for accurate diagnosis and hopefully accurate early diagnosis to SCC.

This work developed a novel method to find small numbers of miRNAs that are able to separate healthy samples from SCC samples with clear and wide margin in 2D or 3D spaces. Our method was tested on the SCC miRNA expression data set from [10]. Many 2-and 3-miRNA groups (together with their specific expression ranges) were discovered as clear linear discriminant rules for the diagnosis of SCC. The basic idea of our method is the construction of an innovative committee of decision trees by using the C4.5 algorithm [36] iteratively. The preprocess of the data involves a prioritization method to rank the whole number of miRNAs and then to focus on potential candidates by projecting wet-lab confirmed plasma and tissue miRNA biomarkers onto this ranked list of miRNAs ordered by miRNAs' gain ratio [37]. This feature selection method is capable of recommending those highly ranked miRNAs not yet studied by wet-labs in the past for rule discovery, and capable of suggesting a good mapping between lung tissue-specific and plasma-specific miRNA biomarkers useful for a minimally invasive diagnosis. For the discovery of the most reliable rules, a distance separation technique is used to determine the Max-Min distance between the normal and cancer classes separated by each rule, and the widest distance is then taken to recommend the best rules. In addition, we also considered a computationally heavy method to detect rules from the whole feature space. We further demonstrated the reliability of these biomarkers by comparing the performance of the most reliable 2-miRNA (3-miRNA) rules with that of 1000 randomly selected 2 miRNAs (3 miRNAs) with C4.5 decision tree classifier and 10-fold cross validation, and performing a resampling test by disordering the class labels.

For all of the miRNAs involved in our 2-miRNA rules, we examined their chromosomal locations and their common target genes. We also established links between the diseases and chromosomal locus with the common target genes to show that most of the chromosomal loci have a high frequency of genomic alteration in lung cancer and that two sets of our biomarkers have confirmed associations with lung cancer.

## Materials and methods

### Data sets of miRNA expressions in SCC patients

Two data sets are used by this work. Data set 1 is a collection of miRNA expressions in SCC tissues which had been studied by [10] for comparative analysis of differentially expressed miRNAs between normal and SCC tissues. Here, it is used for rule discovery. In this data set, there are 61 SCC tissue samples and 10 matched adjacent normal lung tissue samples for the miRNA expression profiling. These samples were collected from patients in the University of Michigan Hospital between October 1991 and July 2002 with patient consent and institutional review board approval. Total RNAs of these 71 samples were preprocessed and then profiled on MirVan miRNA Bioarray (version 2, Ambion) which contains 328 human miRNA probes. So, this data set is a 71 × 328 relational table with each row associating with a class label "cancer" or "normal". The original miRNA expression data was normalized by the quantile and log2 methods, and it was stored at the National Center for Biotechnology Information (NCBI) Gene Expression Omnibus [38] under the accession number GSE16025.

Data set 2 [16] is used as an independent data set to assess the importance of our rules. Data set 2 comprises 187 cancer tissues and 174 adjacent normal tissue from patients described by the expression levels of 549 miRNAs. The expression levels in this data set were processed by subtracting the background as average values of the replicate spots of each miRNA and filtering out the expression signal of faint spots below 600. This data set can be downloaded from the Gene Expression Omnibus under the accession number GSE15008. Since it is impossible to confirm the 34 paired cancerous and adjacent normal samples described by [16] from all the published studies, we are unable to choose this large sample size as the training set.

### Rule discovery within top-ranked miRNAs

We discover simple rules in the form:

(1) $$a_{1}\leq x_{1}\leq b_{1}\cap a_{2}\leq x_{2}\leq b_{2}$$

where *x*_1 _and *x*_2 _represent two miRNAs, [*a*_1_*, b*_1_] is the expression range of *x*_1_, and [*a*_2_*, b*_2_] is the expression range of *x*_2 _(*a*_1 _and *a*_2 _can be −*∞*; *b*_1 _and *b*_2 _can be +*∞*; one of a_*_ and b_*_ must be infinite). If every cancer sample's expression profile satisfies (falls into) the two specific expression ranges, but none of the normal sample profiles satisfies, then we say it is a 100%-frequency rule to differentiate the cancer samples from the normal samples. The complete form of this rule is denoted by

(2) $$a_{1}\leq x_{1}\leq b_{1}\cap a_{2}\leq x_{2}\leq b_{2}\to cancer\left(100\%\right)$$

It can be suggested that if the expression of *x*_1 _is between *a*_1 _and *b*_1 _for a test lung cell sample, and the expression of *x*_2 _is between *a*_2 _and *b*_2_, then this test sample is very likely to be a cancer cell. Similarly in this work, we also define a 100%-frequency rule to differentiate normal samples from cancer samples. Such strong rules can be easily visualized in 2D spaces to facilitate biological interpretation of the computational results.

This work focuses on 2-miRNA or 3-miRNA 100%-frequency rules as biomarkers for the diagnosis of SCC. We do not identify 100%-frequency rules with 4 or 4+ miRNAs. Our rule discovery method is based on decision trees which usually generate rules combining 2 or 3 miRNAs with their specific expression ranges. Decision tree is a classical idea to induce a set of exclusive rules covering the training data only once, and thus the rules are sensitive to slight change of training data. Due to this constraint, using a single decision tree usually loses some prediction accuracy [39].

Our method has two innovative parts. One is a novel idea to generate a committee of decision trees to discover 100%-frequency rules; the other is a simple projection method to narrow down important miRNAs from the 328 miRNAs for the induction of the decision tree ensemble.

As the first step of the projection method, we prioritize and rank the 328 miRNAs in the data set based on their gain ratios over the 71 samples' expression profiles. Gain ratio [37] measures a collective difference of every single miRNA's expressions between the two classes. A high gain ratio indicates that the miRNA is a high-potential biomarker differentially expressed over the two classes. As the second step, we project wet-lab confirmed and intensively studied miRNAs onto this rank list. Using this step, we can recommend those highly ranked miRNAs that have not been studied in wet-labs in the past for rule discovery and potentially for fresh biological study.

In this work, we use 5 plasma biomarkers (miR-486, miR-126, miR-182, miR-210 and miR-21) identified in 28 NSCLC patients including 14 adenocarcinoma and 14 SCC patients [15] for the above rank list projection. All of these miRNAs are confirmed as key biomarkers in early lung cancer diagnosis. These miRNAs in plasma are also a subset of 12 previously identified tissue biomarkers validated by paired SCC tissues and noncancerous tissues associated with early-stage lung cancer [40]. So these 5 miRNAs can serve as a guideline for the next step of tissue-specific biomarkers identification.

**Figure 1:**
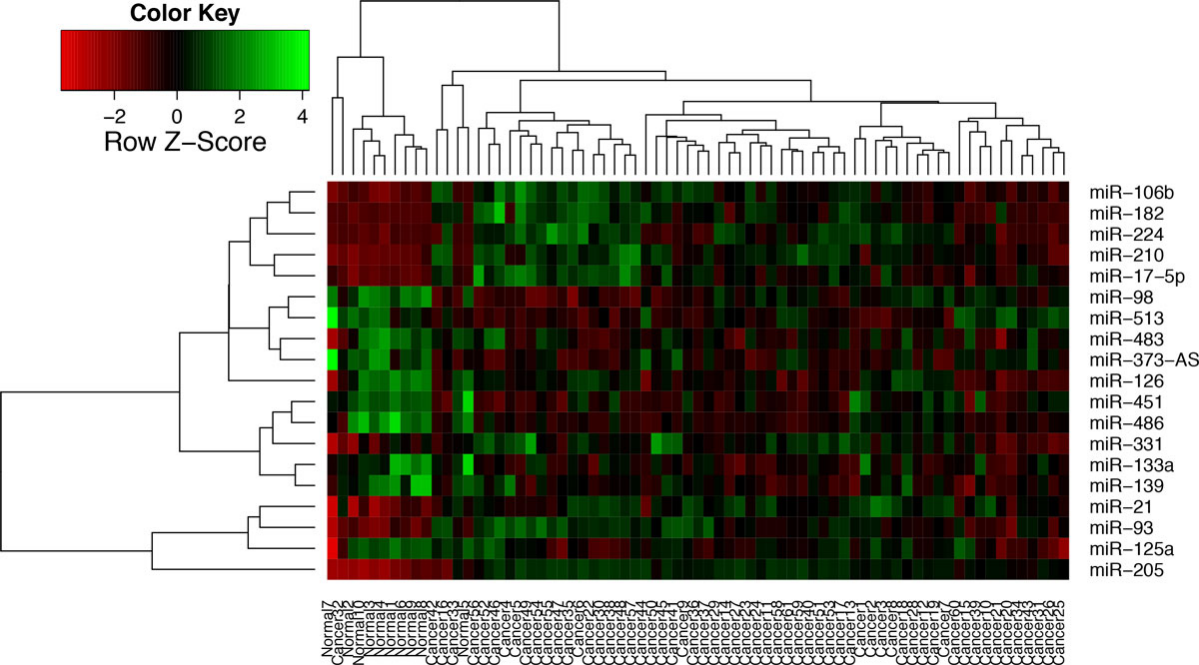
**Heatmap representation of the expression levels of the 19 miRNAs**. A single miRNA is unable to distinguish cancer samples from normal samples, while combining 2 or 3 miRNAs can identify cancer (or normal) samples from the normal (or cancer) samples without mistake.

The projection of the 5 plasma biomarkers against the list of prioritized 328 miRNAs is shown in Table 1. The 5 confirmed miRNAs are mapped to positions 1, 3, 5, 13 and 19. However, none of these 19 individual miRNAs is a good biomarker to separate the two classes of data as shown in Figure 1. So, we concentrate on the entire expression data of these 19 miRNAs to derive groups of miRNAs for 100%-frequency rules. The remaining data (i.e., excluding the 19 miRNAs) is used for comparison to examine the effectiveness of our rule discovery method.

**Table 1:** Projection of 5 important miRNAs onto a prioritized list of 328 miRNAs, resulting in 19.

miRNA	Rank	Expression	P-value	miRNA	Rank	Expression	P-value
**miR-486**	1	Down	3.12e-05	miR-125a	11	Down	8.857e-02

miR-98	2	Down	4.631e-07	miR-93	12	Up	6.401e-06

**miR-126**	3	Down	1.14e-02	**miR-210**	13	Up	5.548e-12

miR-205	4	Up	3.678e-07	miR-224	14	Up	2.866e-14

**miR-182**	5	Up	2.2e-16	miR-17-5p	15	Up	3.646e-11

miR-106b	6	Up	1.224e-09	miR-373-AS	16	Down	3.647e-03

miR-133a	7	Down	4.208e-03	miR-483	17	Down	4.11e-02

miR-513	8	Down	2.263e-02	miR-139	18	Down	3.812e-03

miR-451	9	Down	2.713e-05	**miR-21**	19	Up	1.293e-04

miR-331	10	Up	4.124e-02				

miRNAs ranked as high as these 5 miRNAs

To construct a committee of decision trees for the discovery of multiple 100%-frequency rules, we induce

the first decision tree from the 19-miRNA data set. To induce the second tree, we remove the field (attribute values from the data) of the root node miRNA of the first tree from the data set. Iteratively, we construct a subsequent decision tree by removing the data of the root node miRNA of the current tree. This process continues until there are only two miRNAs left in the data set. We use the R software package [41] and its C4.5 implementation to construct each decision tree (The source code of algorithm constructing a committee of decision trees is described in the Additional file 1).

**Figure 2:**
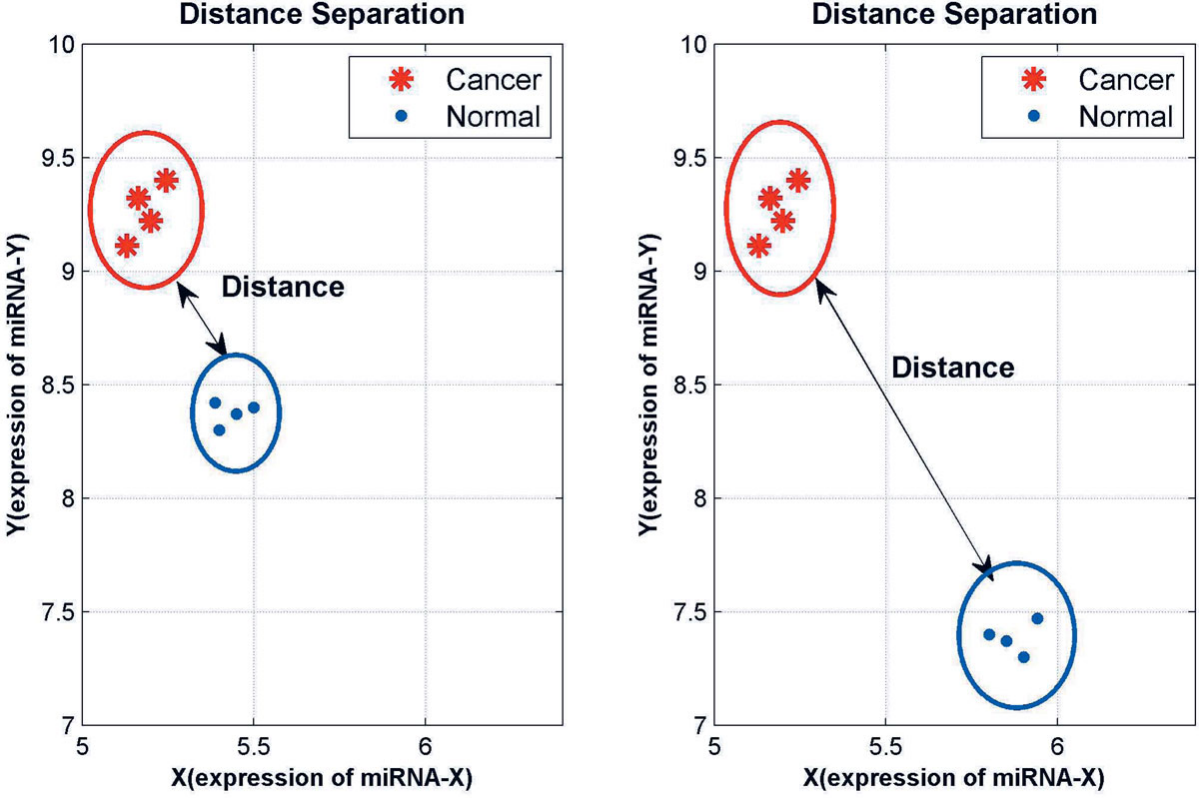
**Distance separation by 100%-frequency rules in 2D space**. The left panel shows a shorter distance separation between the cancer and normal samples than the separation shown in the right panel.

Every 100%-frequency rule with two or three miRNAs can separate the cancer samples clearly from the normal samples in 2D or 3D spaces. As a wider separation suggests a more reliable biomarker rule (Figure 2), we measure the separation extent by using the shortest pair-wise Euclidean distance between the cancer and normal samples. When multiple 100%-frequency rules are generated, further data analysis is on those with a wider separation distance (i.e., the Max-Min distance).

**Figure 3:**
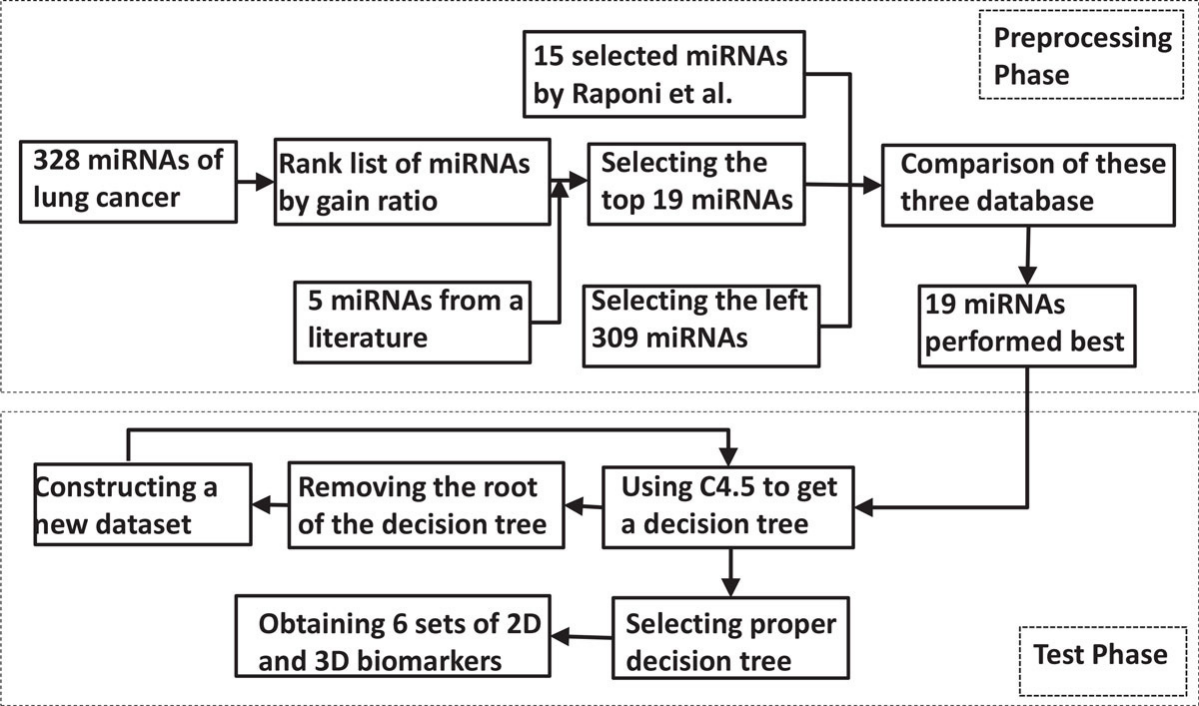
**The procedure of rule discovery with 19 miRNAs**. The up panel is the dataset processing phase, and 19 miRNAs are obtained. The down panel is the discovery phase to get biomarkers.

The entire work flow of our rule discovery method with feature space projection is summarized in Figure 3. The best two or three miRNA biomarkers identified by our method cannot produce an accuracy of 100% by using simple linear discriminant analysis of support vector machine.

### Rule discovery across the whole feature space

Our feature ranking and projection method is good to select important miRNAs to derive 100%-frequency rules. However, some bias may occur as our list of "extensively studied miRNAs in the literature" may be far from complete. To ensure there is less bias, we search the whole feature space, namely across all of the 328 miRNAs, to find strong rules. However, the exploration of every possible combination of these 328 miRNAs leads to exponentially computational cost.

Therefore, our method is restricted to combine all possible 2-and 3-miRNAs and all possible valid expression ranges of these miRNAs to see whether the combined ranges satisfy every cancer sample's expression profile. If this is true, we then examine whether the combined ranges do not satisfy any of the normal samples. If this comes true as well, then the combined expression ranges, together with the miRNAs, form a 100%-frequency rule to distinguish all of the cancer samples from all of the normal samples in 2D or 3D spaces. Similarly, we detect such rules to distinguish 100% of the normal samples from the cancer samples. We also use the distance separation technique to identify more reliable rules.

## Results

Our results are presented in five parts. The first part reports 2-miRNA and 3-miRNA rules and classification performance. The second part is related to distance separation of the rules in 2D or 3D spaces. The third part illustrates the reliability of the identified best miRNA rules. The fourth part presents the chromosomal locations of the miRNAs, and the last part is related to association studies between miRNA biomarkers and disease genes.

### Prediction performance by rules

#### Comparison with literature methods

To show the effectiveness of our feature projection method on prediction accuracy, we compared the prediction performance of three commonly used classifiers on four data sets. One is the data set prepared by [10] which consists of 15 differentially expressed miRNAs extracted from the initial 328 miRNAs. The second data set contains only the 5 plasma miRNAs [15] which we used to project out our top-ranked 19 miRNAs. The third data set is our data set consisting of the 19 top-ranked miRNAs (Table 1). The fourth data set contains all the data after the removal of the third data set (the 19-miRNA data set) from the 328-miRNA data set. Note that there is not much miRNA overlapping between the first and third data set (only 6 miRNAs in common). We used k-nearest neighbor classifier (KNN, k = 1), Naive Bayes (NB), and C4.5 decision tree (C4.5) classifier to conduct the prediction under a 10-fold cross-validation scheme.

Table 2 shows the prediction performance (specificity, sensitivity, F1 measure and ROC area) of the three classifiers on these four data sets. It can be seen that the three classifiers all performed better on the 5-plasma miRNAs data set and on our 19-miRNA data set than on the other two data sets. This indicates that the 5 plasma biomarkers are indeed good biomarkers, and the 19 prioritized and projected miRNAs are indeed good potential candidates for rule discovery and biomarker identification.

**Table 2:** Comparisons of three classifiers on four data sets.

Data sets	Algorithms	Specificity	Sensitivity	F-Measure	ROC Area
15 miRNAs (Raponi et al. 2009)	KNN	0.9833	0.8182	0.975	0.934

	NB	0.9833	0.8182	0.975	0.934

	C4.5	0.9516	0.7778	0.959	0.827

5 miRNAs (Shen et al. 2010)	KNN	0.9839	1.0000	**0.992**	0.944

	NB	0.9839	1.0000	**0.992**	0.989

	C4.5	0.9672	0.8000	**0.967**	0.84

19 miRNAs (top ranked)	KNN	0.9839	1.0000	**0.992**	0.944

	NB	0.9836	0.9000	**0.984**	0.946

	C4.5	0.9524	0.8750	**0.968**	0.798

309 miRNAs (lower ranked)	KNN	0.9833	0.8182	0.975	0.926

	NB	0.8413	0.6250	0.935	0.779

	C4.5	0.8413	0.3846	0.891	0.666

#### Multiple rules derived from the top-ranked 19 miRNAs

We applied C4.5 to our 19 top-ranked miRNAs data set to construct the first decision tree (denoted by DT1). As described in the Method section, we then removed the root node miRNA of DT1 from the data set to construct the second tree (denoted by DT2). By iteration, we constructed a total of 18 decision trees. Interestingly, DT1 does not contain any 100%-frequency rules. In fact, 6 of the 18 decision trees (DT2, DT3, DT4, DT9, DT10, and DT15) produce an accuracy of 100% consisting of 2 or 3 miRNAs. Only three decision trees (DT2, DT3 and DT4) are able to form the 100%-frequency biomarker rules.

**Figure 4:**
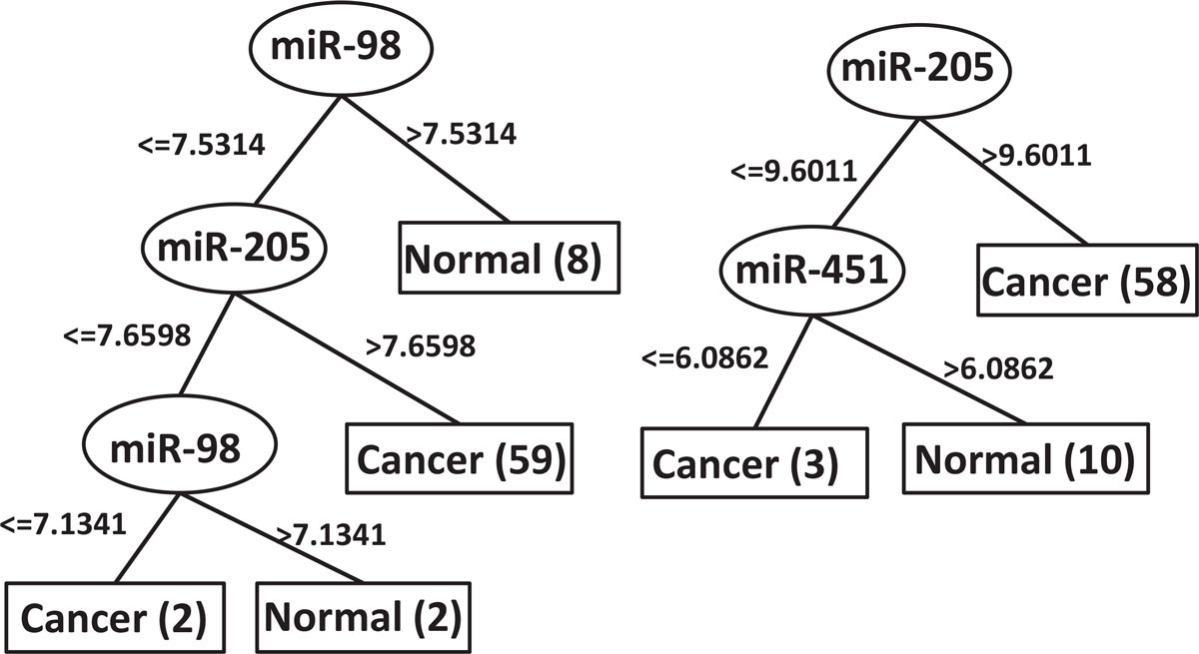
**Decision trees**. The left panel is a decision tree made of miR-205 and miR-98. The right panel is a decision tree made of miR-205 and miR-451.

**Figure 5:**
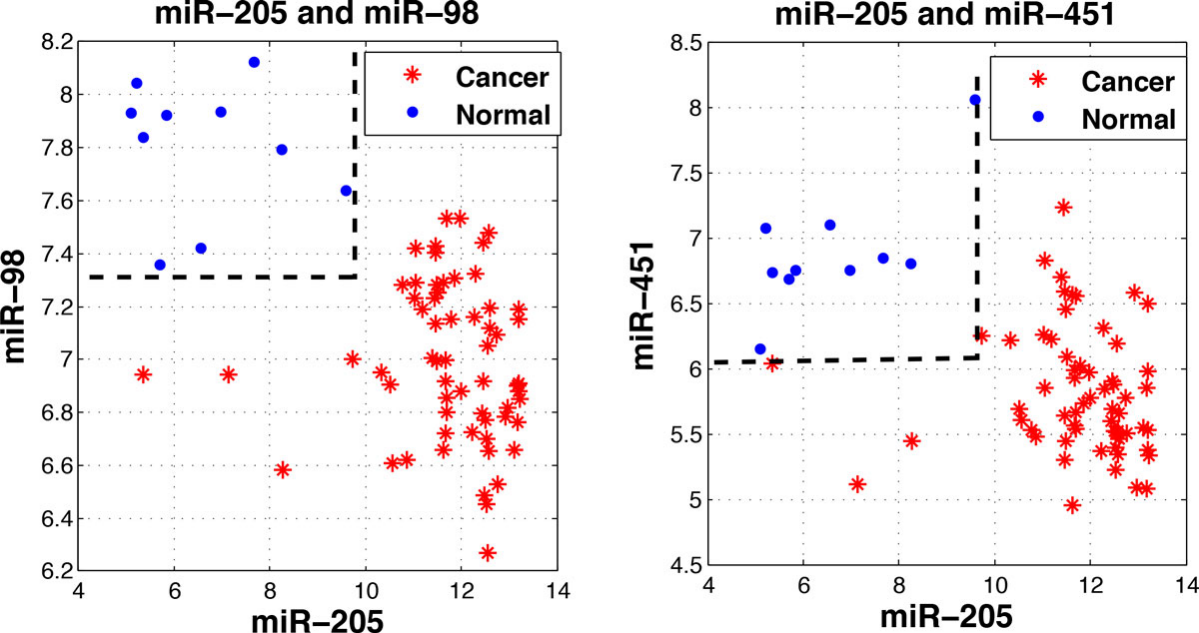
**Expression data on 2D planes**. The left panel is the plane co-ordinated by miR-205 and miR-98. The right panel is coordinated by miR-205 and miR-451. The blue boundaries indicate the expression ranges of all of the normal samples.

As an example, Figure 4 displays the tree structures of DT2 and DT4. Both of them contain only two miRNAs. The 100%-frequency rules derived from these two trees separate the cancer and normal samples in a way as shown in Figure 5 where the x-y axis of the 2D planes represents the expression ranges of these miRNAs.

#### Classification performance under 5-fold training-test experiments

The derived rules above can separate the two classes of samples clearly without any mistake. However, they are derived from the top-ranked miRNAs based on all of the 71 samples. To demonstrate the generalization ability of the rules induced by our method, we conducted C4.5's 5-fold training-test experiments. The initial 10 normal samples and 61 cancerous samples are randomly divided into 5 parts. Four parts of the data set were used as a training data set, and 5 training data sets were constructed (TrS1, TrS2, TrS3, TrS4 with 57 samples, and TrS5 with 56 samples). Correspondingly, the remaining part was reserved as a test data set, and 5 test data sets were constructed (TeS1, TeS2, TeS3, TeS4 with 14 samples, and TeS5 with 15 samples, each containing two normal samples). By our method, the gain ratio and the 5 plasma miRNAs projection method were applied to select miRNAs from the 5 training sets. Actually we obtained 27, 21, 14, 32, and 20 top-ranked miRNAs respectively. Then the rules were derived within these top-ranked miRNAs and the Max-min distance step was applied to determine the most reliable rule. The TrS1, TrS2, TrS4, and TrS5 training data sets have the same best rule (made from miR-205 and miR-451), while the TrS3 has the rule made from miR-205 and miR-21. Finally, we applied these reliable rules to the corresponding test sets, and all achieved an accuracy of 100%, except TeS4 with 92.86% (1 cancer sample misclassified). The details are described in Additional file 2.

#### Assessing the importance of miRNA biomarkers by using an independent data set

Data set 2 [16] contains miRNA expression data of 187 cancer tissues and 174 adjacent normal tissue from patients. The platform for generating data set 2 (the National Engineering Research Center mammalian microRNA microarray with 549 human miRNAs) is different from the platform of data set 1 (MirVan miRNA Bioarray, version 2). The two data sets are preprocessed by different methods as well. Because of these differences, it is not reasonable to directly test the miRNA expression ranges on data set 2 for a rule derived from data set 1. However, the miRNAs in a rule of data set 1 can be still validated on the data set 2 by testing whether these miRNAs are able to classify the samples in data set 2 with a high accuracy. A high classification performance would suggest that these miRNAs are robust across different data sets and thus they are worth of further investigation. We note that the miRNAs in a rule from data set 1 is detected independently from data set 2.

To test whether these miRNA biomarkers discovered from data set 1 have a good generalization ability, we carried out 10-fold cross-validation on the expression data of only these miRNAs of data set 2 (the independent data set) to see the classification performance in C4.5. We compared the sensitivity, specificity, accuracy, ROC area and F-measure for three data sets: data set 2 of 549 miRNAs, the data set of top-ranked 158 miRNAs, and the data set of 3 miRNAs (miR-126, miR-205 and miR-182) which are from the best rule from data set 1 (with the largest distance 0.7799). The classification performance on these three data sets are shown in Table 3. We can see that the classification using just the 3 miRNAs from the best rule of data set 1 achieved an accuracy of 84.49%, sensitivity of 91.40% and specificity of 77.14%. This performance is better than the classification performance by using all miRNAs in data set 2. Although the specificity decreases, the cost in real-life diagnostic would be lower using the just 3 miRNAs, because the cost of misclassifying 'normal' as 'cancer' is much smaller than misclassifying 'cancer' as 'normal'. These results demonstrate that the miRNA biomarkers identified from data set 1 are also biomarkers to separate the two classes of samples in the independent data set 2 with a high accuracy. This implies that our miRNA biomarkers have a good generalization ability in classification.

**Table 3:** The performance comparison of three datasets.

Data sets	Sensitivity	Specificity	Accuracy	ROC area	F-measure
549 miRNAs	0.8441	0.8343	0.8393	0.817	0.844

158 miRNAs	0.8656	0.8111	0.8393	0.845	0.847

3 miRNAs	0.9140	0.7714	0.8449	0.853	0.859

#### Rules derived by using the whole feature space

**Figure 6:**
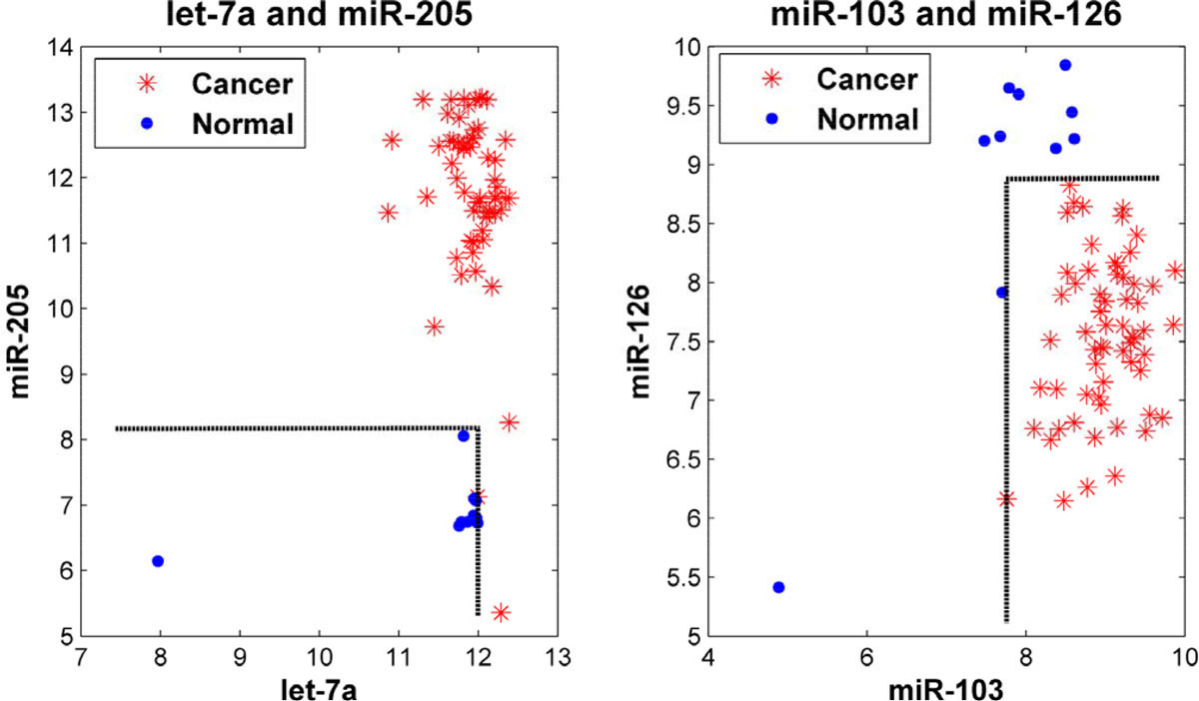
**Examples of 2-miRNA rules**. The left panel describes two miRNAs whose class-label is related with normal. The right panel shows two miRNAs whose class-label is related to cancer.

**Figure 7:**
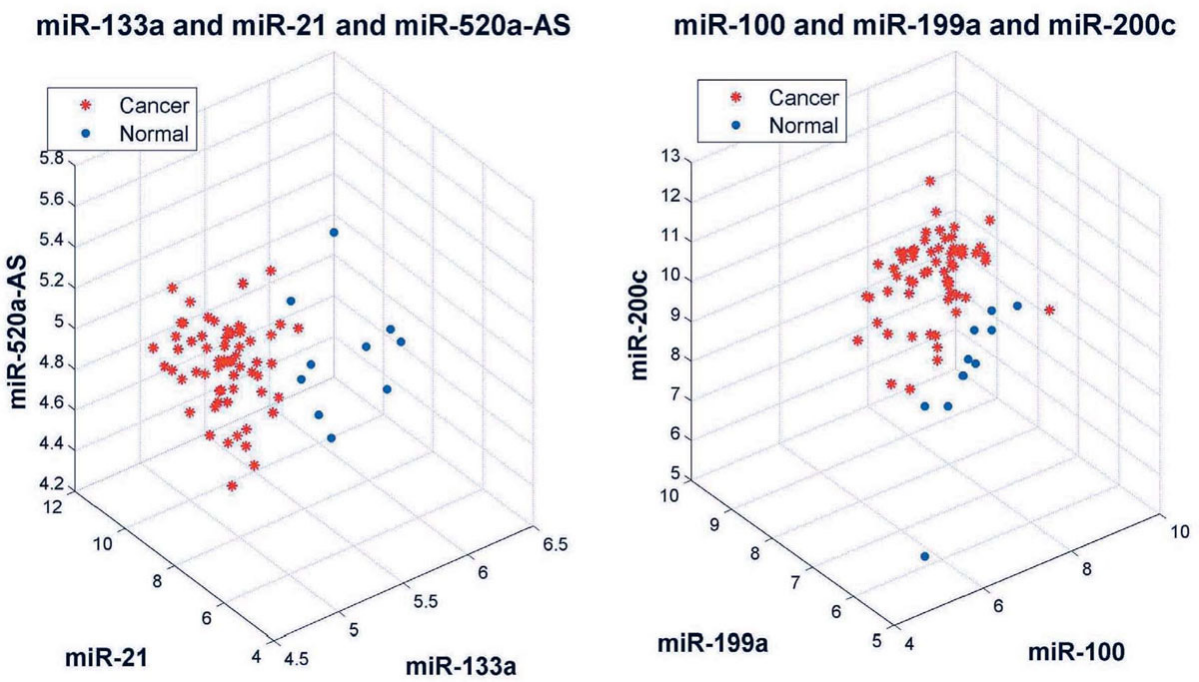
**Examples of 3-miRNA rules**. The left panel contains miR-100, miR-199a and miR-200c. The right panel contains miR-133a, miR-21 and miR-520a-AS.

On the whole feature space, our rule mining method detected a total of 14 new 100%-frequency rules each of which combines only two or three miRNAs, in addition to the 3 rules identified by the decision tree committee. Two of them are displayed in Figure 6. The rules are: −*∞ *<*let *− 7*a *≤ 11.989 ∩ −*∞ *<*miR *− 205 ≤ 9.601 → *Normal*(100%) ( −*∞ *and +*∞ *can be omitted.); 7.755 ≤ *miR *− 103 < +*∞ *∩ −*∞ *<*miR *− 126 ≤ 8.825 → *Cancer*(100%). Again, it can be seen that these two sets of biomarkers are able to distinguish the 71 cancer and normal samples with no mistake. Examples of 3-miRNA 100%-frequency rules are shown in Figure 7. The rules are: −*∞ *<*miR *− 133*a *≤ 5.844 ∩ 7.381 ≤ *miR *− 21 < +*∞ *∩ −*∞ *<*miR *− 520*a *− *AS *≤ 5.229 → *Cancer*(100%); −*∞ *<*miR *− 100 ≤ 8.706 ∩ −*∞ *<*miR *− 199*a *≤ 7.091 ∩ −*∞ *<*miR *− 200*c *≤ 9.890 → *N ormal*(100%).

### Distance separation in 2D and 3D spaces to identify reliable biomarkers

We calculated the Euclidean distance for the rules discovered from the whole data set 1 (i.e., the 71 samples), and used the shortest pair-wise distance and the Max-Min technique to identify the best miRNA biomarkers (Table 4). In our method, we selected top × significant rules, and × is the ceiling of the 1/3 of the total number of rules. Therefore, we selected top four 2-miRNA rules with the distance cut-off threshold 0.20 and top three 3-mIRNA rules with the cut-off threshold 0.45.

**Table 4:** Shortest pair-wise Euclidean distance between the cancer and normal samples in 2-miRNA and 3-miRNA biomarker spaces.

Biomarker	miRNAs in the Rules	Shortest Distance	Rank
2-miRNA biomarker	**miR-205 and miR-98**	**0.5421**	**2D.1**

	**miR-205 and miR-451**	**0.4311**	**2D.2**

	**miR-103 and miR-126**	**0.3591**	**2D.3**

	**let-7a and miR-205**	**0.2496**	**2D.4**

	miR-210 and miR-98	0.1892	2D.5

	miR-137 and miR-98	0.1660	2D.6

	miR-106b and miR-29b	0.1498	2D.7

	miR-17-5p and miR-451	0.1398	2D.8

	miR-149 and miR-182	0.0941	2D.9

	miR-324-3p and miR-43	0.0879	2D.10

	let-7b and miR-486	0.0835	2D.11

3-miRNA biomarker	**miR-126, miR-205 and miR-182**	**0.7799**	**3D.1**

	**miR-100, miR-199a and miR-200c**	**0.7275**	**3D.2**

	**miR-133a, miR-21 and miR-520a-AS**	**0.4515**	**3D.3**

	miR-133b, miR-139 and miR-210	0.2459	3D.4

	miR-1, miR-106a and miR-203	0.1589	3D.5

	let-7i, miR-130a and miR-224	0.1231	3D.6

From Table 4, it can be seen that miR-205 and miR-98 constitute our best 2-miRNA rule that

(3) $$7.356\leq miR-98<+\infty \cap -\infty <miR-205\leq 9.601\to Normal\left(100\%\right)$$

for the diagnosis of SCC. In fact, this rule separates the normal and cancer classes with a distance of at least 0.5421 in 2D space. Their chromosomal locations, common target genes, and associations with disease genes are presented in later part.

Classification performance on the data of only these two miRNAs was also evaluated. The performance (F1 Measure: KNN-1.000, NB-0.984, C4.5-0.976) is higher than that on the 19-miRNA data set, or on the 15-miRNA data set (Table 2).

The other three important 2-miRNA rules are formed by miR-205 and miR-451, by miR-103 and miR-126, or by let-7a and miR-205. The best 3-miRNA rule is formed by miR-126, miR-205 and miR-182; the second best is by miR-100, miR-199a and miR-200c; and the third best is by miR-133a, miR-21 and miR-520a-AS. Table 5 shows the details of the rules of these best 2-and 3-miRNA biomarkers including their expression ranges.

**Table 5:** Important 2-miRNA and 3-miRNA rules by using the shortest pair-wise distance and the Max-Min technique.

miRNA biomarkers	Their expression ranges and the rules
miR-98 and miR-205	7.356 ≤ *miR *− 98 < +*∞ *∩ −*∞ *<*miR *− 205 ≤ 9.601 → *N ormal*(100%)

miR-451 and miR-205	6.148 ≤ *miR *− 451 < +*∞ *∩ −*∞ *<*miR *− 205 ≤ 9.601 → *N ormal*(100%)

let-7a and miR-205	−*∞ *<*let *− 7*a *≤ 11.989 ∩ −*∞ *<*miR *− 205 ≤ 9.601 → *N ormal*(100%)

miR-103 and miR-126	7.755 ≤ *miR *− 103 < +*∞ *∩ −*∞ *<*miR *− 126 ≤ 8.825 → *Cancer*(100%)

miR-126, miR-205 and miR-182	−*∞ *<*miR *− 126 ≤ 8.825 ∩ 5.354 ≤ *miR *− 205 < +*∞ *∩5.551 ≤ *miR *− 182 < +*∞ *→ *Cancer*(100%)

miR-133a, miR-21 and miR-520a-AS	−*∞ *<*miR *− 133*a *≤ 5.844 ∩ 7.381 ≤ *miR *− 21 < +*∞ *∩ − *∞ *<*miR *− 520*a *− *AS *≤ 5.229 → *Cancer*(100%)

miR-100, miR-199a and miR-200c	−*∞ *<*miR *− 100 ≤ 8.706 ∩ −*∞ *<*miR *− 199*a *≤ 7.091 ∩ − *∞ *<*miR *− 200*c *≤ 9.890 → *N ormal*(100%)

### The reliability of identified best 2-miRNA and 3-miRNA biomarkers

We applied 10-fold cross-validation test on the best 2-miNRA (miR-205 and miR-98) and 3-miRNA rules (miR-126, miR-205 and miR-182) to see the classification performance by C4.5 (R package RWeka). We further performed a randomization test to see whether the best 2-miRNA (or 3-miRNA) miRNAs are better predictors than randomly selected 2 miRNAs (or 3 miRNAs). The random selection was repeated 1000 times. All the area under ROC curves (AUCs) were calculated and compared. The best 2-miNRA rule had an average AUC = 1.0 in the 10-fold cross-validation, and the best 3-miRNA rule had an average AUC = 0.9975. For the randomly selected 2 miRNAs, only a probability of 0.007 could produce an AUC*≥*0.999 for the 1000 repeated tests. For the randomly selected 3 miRNAs, only a probability of 0.012 could produce an AUC*≥*0.9975. The probabilities in different AUC scales are shown in Table 6. These results indicate that our miRNA biomarkers are significant and reliable, instead of random. We further performed a resampling test by disordering the class labels, and no rules were found using our method.

**Table 6:** The probability of different AUC values in the 1000 randomization tests.

2-miRNA AUCs	Probability	3-miRNA AUCs	Probability
*≥ *0.9	0.177	*≥ *0.9	0.328

*≥ *0.95	0.089	*≥ *0.95	0.19

*≥ *0.98	0.035	*≥ *0.98	0.091

*≥ *0.99	0.025	*≥ *0.99	0.062

*≥ *0.998	0.009	*≥ *0.9975	0.02

*≥ *0.999	0.007	*≥ *0.999	0.012

### The genomic location of biomarker miRNAs

Many known human miRNAs reside in particular genomic regions that are prone to alteration in cancer cells. For example, the main chromosomal alteration loci of miR-15 and miR-16 are identified at 13q14 with down-regulation, which is the first association study between miRNA genes and cancer [42,43]. We obtained the chromosomal locations of all of the 13 miRNAs in the 100%-frequency rules of a wide separation in 2D and 3D spaces (the 7 top-ranked rules in Table 4). This location information was obtained through keyword search from the miRNAMap database [44] and miRBase database [45-47]. For the miRNAs let-7a, miR-133a and miR-199a, we obtained three loci for each of them. Details are presented in Table 7.

**Table 7:** The chromosomal location of the 13 miRNAs in our 2D and 3D biomarker rules.

miRNAs	Chr location	miRNAs	Chr location
let-7a-1,-2,-3	9q22.2,11q24.1, 22q13.3	miR-199a-1,-2	19p13.2, 1q23.2

		miR-133a-1,-2	18q11.1,20q13.3

miR-21	17q23.2	miR-200c	12p13.31

**miR-98**	**Xp11.2**	**miR-205**	**1q32.2**

miR-100	11q24.1	miR-451	17q11.2

miR-126	9q34	miR-520a-AS	19q13.42

It has been previously reported that there are many chromosomal arms having frequent loss of heterozygosity [48], such as 1p, 3p, 4p, 4q, 5q, 8p, 9p (p16), 9q, 10p, 10q, 13q (Rb), 15q, 17p (p53), 18q, 19p, Xp, and Xq, in frequency order for lung cancer [9,45,46,49]. In this study, we identified some new chromosomal arms such as 11q, 22q, 17q, 20q, 1q and 12p. In particular, the best 2-miRNA rule biomarkers miR-98 and miR-205 are located at Xp11.2 and the new arm 1q32.2. In fact, these two arms have been studied before for various purposes. It was reported by [50] that there are 5 cases of renal cell carcinoma with translocation involving Xp11.2 in children. It was found by [51] that chromosome 1q32.2, based on an alignment of the mature miR-205, controlled epithelial-to-mesenchymal transition. It was also claimed by [52] that renal cell carcinomas are associated with Xp11.2 translocation in five adult patients. Sham *et al*. [53] identified several nonrandom chromosomal changes in 31 primary ovarian carcinomas in Chinese women, including gains of 1q (10 cases, 32%), and that the losses of 1q32.2 were observed as alterations in comparative genomic hybridization studies. These results showing the alterations of these two locations in cancers support our suggestion that combining miR-98 and miR-205 is a good approach to lung cancer study.

### Target genes of biomarker miRNAs and their associated diseases

For each 100%-frequency rule containing 2 or 3 miRNAs, we detected target mRNAs of these miRNAs. Then we identified their common targets. From these common targets, we also linked to the OMIM disease database to examine disease gene information.

The target genes of the miRNAs in the 4 top-ranked 2-miRNA rules (Table 4) were extracted from the

TargetscanHuman database [54,55]. All of them have many target genes. For example, miR-451, -126, -98, -205, -103 and let-7a have 20, 25, 46, 415, 531 and 84 target genes respectively. Then we looked at the common target genes of the miRNAs involved in one rule. Interestingly, the common targets are not many. For example, miR-98 and miR-205 have only two common targets FZD3 and RPS6KA3. Details are shown in Table 8.

**Table 8:** The targets and associated diseases of our biomarkers.

Biomarkers	Common targets	OMIM gene/ disorder	Relate to lung cancer or carcinoma
**miR-98 and** **miR-205**	FZD3 RPS6KA3	606143/- 300075/303600	**carcinoma** **squamous cell carcinoma**

miR-451 and miR-205	AEBP2	-/-	irrelevant

let-7a and miR-205	PARD6B NKD1 MAP3K2 RBMS2 EPB41	608975/- 607851/- 609487/- 602387/- 130500/61804	irrelevant **lung cancer** irrelevant irrelevant **lung cancer**

miR-103 and miR-126	AKAP13	604686/-	irrelevant

The first and third top-ranked miRNA pairs (Table 8) have opposite change of expression in normal samples compared to the disease samples. These pairs of miRNA may effect different complementary pathways. It is possible that the down regulated miRNA inhibited a transcription factor that regulates the other miRNA. On the other hand, the common targets of the pairs of miRNAs are sensible only when (i) down-regulation of their common targets causes cancers, and (ii) their common targets have normal or high expression in normal tissues. For example, NKD1, FZD2 and EPB41 fit the biological behavior expected above. Especially, down regulation of NKD1 (common target of let-7a and miR-205) increases invasive potential of NSCLC [56]. FZD3 works the same way ("The proliferation and invasion ability of SACC-M cells were enhanced when the expressions of FZD2 and FZD3 genes were inhibited in SACC-M cells") [57]. EPB41 (common target of let-7a and miR-205) is another example that works this way. It is absent in most NSCLC cancer. Its presence suppresses these lung cancer cells' growth [58].

From these target genes, we further conducted disease gene analysis. First, we obtained the common target genes' OMIM information and their associated diseases from Human Disease Gene List [59] with the target genes' name. To this end, we compared the associated diseases of these biomarkers. It was found that: (i) the two miRNAs (miR-98 and miR-205) involved in our best rule have been both confirmed to associate with carcinoma; and (ii) Let-7a and miR-205 (in the second best rule) have been confirmed to be directly associated with lung cancer. On the other hand, we did not find evidence in the literature to show the pair miR-451 and miR-205, or the pair miR-103 and miR-126 linked to lung cancer in any way (Table 8). In addition, from the miR2Disease [60], a manually various human diseases, the five miRNAs (miR-98, miR-205, miR-451, miR-126 and let-7a) have been found to be associated with lung cancer.

## Discussion

As described, this work applied a new rule discovery and distance separation technique to discover 2-miRNA and 3-miRNA 100%-frequency rules for lung SCC diagnosis. We constructed a data set consisting of 19 important miRNAs by projecting 5 plasma miRNA biomarkers onto the whole list of 328 miRNAs ordered by gain ratio. Classification performance on this data set is better than on other data sets. This study can also provide knowledge for us to develop potential non-invasive or minimally invasive diagnostic biomarkers for early lung cancer diagnosis. Of the 5 previously intensively studied plasma miRNAs, three of them (miR-21, miR-126 and miR-182) have been considered to form our diagnostic rules for lung tissue diagnosis. So, these 2-miRNA and 3-miRNA rules and the corresponding miRNAs identified from the tumor tissues may be good plasma miRNA biomarkers as well.

The present study suggests that a minimal 2-miRNA or 3-miRNA rule can distinguish lung SCC tissues from normal tissues. These rules are entirely new, because complex diseases are often affected by various miRNAs rather than a single miRNA, and single-miRNA rules are insufficient for accurate diagnosis.

The advantage of the method presented here can be extended to the study of biomarkers identification in lung cancer prognosis. Also, we can validate the prognostic utility of these identified diagnostic biomarkers in early lung cancer. In addition, the discovered rules and distance separation technique would potentially be applied to further investigation of biomarkers in other cancer diagnosis and prognosis, including breast cancer, pancreatic cancer, etc.

## Conclusions

Rule discovery followed by distance separation is a powerful computational method for reliable identification of miRNA biomarkers. The visualization of the rules and the clear separation between the normal and cancer samples by our rules will help biology experts for their analysis and biological interpretation.

This work has illustrated computational difficulties of multi-miRNA analysis of expression data, and

presented our effective approach to 2-miRNA or 3-miRNA biomarker discovery for lung SCC diagnosis. We proposed a novel method to construct a committee of decision trees which are subsequently used to derive 100%-frequency rules containing 2 or 3 miRNAs. To detect more reliable rules, we applied a Max-Min distance separation technique to look for the clear boundaries between the normal and cancer sample groups. The chromosomal loci of the miRNAs in these rules are identified, and the target genes of these biomarker miRNAs are also obtained from databases to determine the common mRNAs. These common target genes are then linked to diseases. As future work, the proposed method can be applied for plasma biomarkers identification, and it can be taken for diagnosis and prognosis studies related to other cancers.

## Abbreviations

SCC: Squamous cell carcinoma; OMIM: Online Mendelian Inheritance in Man; NSCLC: Non-small cell lung cancer; GEO: Gene expression omnibus, NCBI: National Center for Biotechnology Information; KNN: K-nearest neighbor; NB: Naive bayes; C4.5: C4.5 decision tree; ROC: Receiver operating characteristic; AUCs: Area under ROC curves; DT: Decision tree; TrS: Training set; TeS: Test set.

## Competing interests

The authors declare that they have no competing interests.

## Authors' contributions

RS carried out the experiments and drafted the initial manuscript. QL contributed to the design of the algorithms. GH and HN contributed to the interpretation of the results. LW and KR provided critical comments and important suggestions to revise the work. JL initiated and supervised the study, and revised the manuscript. All authors read and approved the final manuscript.

## Supplementary Material

Additional file 1 **Information of gain ratio and source code of algorithm constructing a committee of decision trees**. Click here for file

Additional file 2 **Summary of an initial data set and the 5-fold training-test experimental data sets**. Click here for file

## Declarations

Publication charges for this work were funded by ARC DP130102124.

This article has been published as part of *BMC Genomics *Volume 15 Supplement 9, 2014: Thirteenth International Conference on Bioinformatics (InCoB2014): Computational Biology. The full contents of the supplement are available online at http://www.biomedcentral.com/bmcgenomics/supplements/15/S9.
