# Supplementary material for: Rule discovery and distance separation to detect reliable miRNA biomarkers for the diagnosis of lung squamous cell carcinoma
Source: BMC Genomics. 2014 Dec 8;15(Suppl 9):S16. doi: 10.1186/1471-2164-15-S9-S16 (PMC4290601; doi:10.1186/1471-2164-15-S9-S16)
Supplement: Additional file 1 — Information of gain ratio and source code of algorithm constructing a committee of decision trees. [file 1471-2164-15-S9-S16-S1.pdf]

Rule discovery and distance separation to detect reliable miRNA biomarkers for the diagnosis of lung squamous cell carcinoma --- supplementary file

Renhua Song[1], Qian Liu[1], Gyorgy Hutvagner[2], Hung Nguyen[2], Kotagiri Ramamohanarao[3], Limsoon Wong[4], Jinyan Li[1*]

[1]Advanced Analytics Institute, Faculty of Engineering and IT, University of Technology, Sydney, NSW 2007, Australia.

[2]Centre for Health Technologies, Faculty of Engineering and IT, University of Technology, Sydney, NSW 2007, Australia.

[3]Dept. of Computing and Information Systems, the University of Melbourne, Vic 3010, Australia.

[4]School of Computing, National University of Singapore, Singapore 117417, Singapore.

Experiment Information

Information gain ratio. In this work, we employed information gain ratio [25]. Let Attr be the set of all attributes and Ex the set of all training examples, value(x, a) defines the value of a specific example x for attribute a, where $x \in EX$, and $a \in Attr$ and $H(x) = E[\log(2, 1/p(x_i))] = -\sum p(x_i)\log(2, p(x_i))(i = 1,2,\cdots n)$ specifies the entropy. The information gain for attribute $a \in Attr$ is defined as follows:

$$IG(Ex,a) = H(Ex) - \sum_{v \in values(a)} \frac{|\{x \in Ex \mid value(x,a) = v\}|}{|Ex|} . H(\{x \in Ex \mid value(x,a) = v\}) \quad (1)$$

The information gain is equal to the total entropy for an attribute if for each of the attribute values a unique classification can be made for the result attribute. In this case the relative entropies subtracted from the total entropy are 0. The intrinsic value for a test is defined as follows:

$$IV(Ex,a) = -\sum_{v \in value(a)} \frac{|\{x \in Ex \mid value(x,a) = v\}|}{|Ex|} * \log_2\left(\frac{|\{x \in Ex \mid value(x,a) = v\}|}{|Ex|}\right) \quad (2)$$

The information gain ratio is just the ratio between the information gain and the intrinsic value:

$$IGR(Ex,a) = IG / IV \quad (3)$$

Information gain ratio biases the decision tree against considering attributes with a large number of distinct values. So it solves the drawback of information gain—namely, information gain applied to attributes that can take on a large number of distinct values might learn the training set too well.

Calculation of Euclidean distance. The Euclidean distance or Euclidean metric is the “ordinary” distance between two points that one would measure with a ruler, and is given by the Pythagorean formula [16, 17]. The Euclidean distance between point p and q is the length of the line segment connecting them.

In Cartesian coordinates, if $p = (p_1, p_2 \ldots p_n)$ and $q = (q_1, q_2 \ldots q_n)$ are two points in Euclidean n-space, then the distance from p to q, or from q to p is given by:

$$d(p,q) = d(q,p) = \sqrt{(q_1 - p_1)^2 + (q_2 - p_2)^2 + \ldots + (q_n - p_n)^2} = \sqrt{\sum_{i=1}^{n} (q_i - p_i)^2}. \quad (4)$$

In this work, we just use two dimensions to calculate the distance, so p= (p1, p2) and q= (q1, q2) then the distance is given by

$$d(p,q) = \sqrt{(p_1 - q_1)^2 + (p_2 - q_2)^2}. \quad (5)$$

Source code of the algorithm constructing a committee of decision trees

Input: data.txt, a 71x329 relational table.

Output: DTc, a committee of 2- or 3- miRNAs' decision trees with 100% accuracy; nrule, the number of selected decision trees; myoutput.txt, all the contents printed in the screen; mygraphs.pdf, pictures of all the selected decision trees.

```
sink("myoutput.txt",append=TRUE, split=TRUE); # save the contents of the screen

pdf("mygraphs.pdf"); # save the output pictures

rm(list=ls());

library(RWeka); #load RWeka

library(stringr); #load String

library(gplots); #load Plot


d<-read.table("data.txt",sep="\t"); # load data from a file

g<-GainRatioAttributeEval(V329~.,data=d); # rank the miRNAs by gain ratio

t=19; # choose the top-ranked 19 miRNAs after mapping the 5 plasma biomarkers

i<-order(g,decreasing=T)[1:t];

i<-c(i,length(d)) # add the column of class label to the new dataset

n<-d[,i]; # obtain a new dataset

nrule<-0; # number of rules

q<-length(i)-1; # the times of constructiong decisions

for (c in 1:q){ # the procedure continues unitll only two miRNAs letf

        DTc<-J48(V329~.,data=n); # use C4.5 to construct a decision tree

        bc<-summary(DTc)$details["pctCorrect"][[1]]; # the accuracy of the decision tree

# JUSTIFY THE ACCURACY OF THE DECISION TREE

        if(bc==100) # if the accuracy equals 100%, then print and draw the decision tree

        {

                # JUSTIFY THE NUMBER OF NODES IN THE DECISION TREE

                str<-DTc$classifier$toString(); # the string structure of the decision tree

                str1<-strsplit(str," <"); # split the string, str1 is a list

                str2<-unlist(str1); # transfer a list to a character

                l<-length(str2); # the length of the character

                id<-seq(1,328,1); id=0;
```

```
        for (i in 1:(l-1)){

                st<-str2[i];

                ll<-nchar(st,type="chars",allowNA=FALSE); # the length of a character

                nst<-substr(st,ll-3,ll); # choose the last four chars

                nst1<-strsplit(nst,"V"); # separate the character in V

                num<-nst1[[1]][2];

                num<-as.numeric(num);

                id[i]=num; # the ID of node in the decision tree

        }


        node<-length(unique(id)); # the number of nodes in the decision tree

        if (node<4){

                plot(DTc);

                nrule<-nrule+1;

        }



}


dtcstr<-DTc$classifier$toString(); # select the root of a decision tree

s1<-strsplit(dtcstr,"J48 pruned tree\n------------------\n\n");

s2<-strsplit(s1[[1]][2]," <",);

s3<-s2[[1]][1];

#s<-as.numeric(s3);

#s3<-str_trim(s3);

if(is.na(s3)=="TRUE"){

        n[,-1];

        }

else{


        n[,eval(s3)]<-NULL; # remove the column of the root

}
```

```
}

print(sprintf('The number of selected decision trees is %d', nrule));
sink();
dev.off();
```
